# Adaptation of thoracic and lumbar curvature and spinal muscle activity under changing gravity

**DOI:** 10.3389/fphys.2025.1549249

**Published:** 2025-05-20

**Authors:** Anita Meinke, Alessandro Longhi Baez, Niklas Wiesmann, Oliver Ullrich, David A. Green, Marcel Egli, Jaap Swanenburg

**Affiliations:** ^1^ Faculty of Medicine, Institute of Aerospace Medicine, University of Zurich, Zurich, Switzerland; ^2^ Faculty of Medicine, Institute of Anatomy, University of Zurich, Zurich, Switzerland; ^3^ Faculty of Biomedical Sciences, Università della Svizzera Italiana (USI), Lugano, Switzerland; ^4^ Centre of Human and Applied Physiological Sciences (CHAPS), King’s College London, London, United Kingdom; ^5^ Institute for Risk and Disaster Reduction (IRDR), University College London, London, United Kingdom; ^6^ Institute of Medical Engineering, Space Biology Group, Luzerne University of Applied Science and Arts, Luzern, Switzerland; ^7^ Integrative Spinal Research, Department of Chiropractic Medicine, Balgrist University Hospital, University of Zurich, Zurich, Switzerland

**Keywords:** spine, curvature, lumbar, micro-gravity, hyper-gravity, parabolic flight

## Abstract

**Introduction:**

The effect of micro-gravity on the lumbar and in particular thoracic regions is poorly understood. The aim of this study was to evaluate spinal curvature across the lumbar and thoracic region, and extensor muscle activity during acute micro-gravity and hyper-gravity induced by parabolic flight. In addition, the association between our proxy measure of spinal curvature, and extensor muscle activity in micro-gravity was investigated.

**Methods:**

During two ESA parabolic flight campaigns, 18 participants (8 female; 33 ± 11 years) were measured under earth-gravity, micro-gravity and hyper-gravity conditions. Spinal curvature was assessed using “spinal curvature backpacks” equipped with 15 laser distance sensors to measure the distance between the backpack and the subject’s back. Change in the area enclosed between the back and the backpack was used to measure change in spinal curvature. Muscle activity of the erector spinae (in 4 locations) and multifidus muscles (1 location) was assessed using surface electromyography transmitters. In addition, the spearmen correlation between muscle activity and spinal curvature in micro-gravity was investigated.

**Results:**

Spinal flattening was observed during micro-gravity exposure, with changes most pronounced in the upper lumbar and lower thoracic spine. Mean-normalized area between the back and backpack decreased significantly in micro-gravity compared to earth-gravity (p = 0.001), but not during hyper-gravity (p = 1.00). The erector spinae responded heterogeneously to different gravity conditions across different assessment sites. Multifidus activity at L5 and erector spinae activity at L4 significantly decreased in micro-gravity compared to earth-gravity and hyper-gravity (p’s <= 0.01) and correlated with spinal flattening ( ρ = 0.69, p = 0.004; ρ = 0.67, p = 0.030).

**Discussion/Conclusion:**

Parabolic flight-induced gravity changes caused upper lumbar and lower thoracic spine flattening in micro-g, while spinal curvature remained unchanged in hyper-g. In micro-g, Multifidus (L5) and Erector Spinae (L4) activity decreased, while in hyper-g, increased ES activity was observed at the upper middle transmitter. The maintained curvature and targeted muscle activation in hyper-g demonstrate protective mechanisms against increased axial loading, crucial for posture and injury prevention in both terrestrial and space environments. The spinal and muscular changes in micro-g indicate the need for targeted countermeasures during spaceflight, warranting comprehensive assessment in future research.

**Ethics:**

French “EST-III” (Nr-ID-RCB: 2022-A01696-37).

## 1 Introduction

The human spine is designed to withstand the force of Earth’s gravity (earth-g), playing a crucial role in supporting the head, neck, and torso when upright. This is evidenced by the spine’s normal curvature, which optimizes balance and minimizes strain. However, the spine’s structure and function undergo notable changes during a prolonged space mission, in the absence of earth-g (Green and Scott, 2018). These changes, which include trunk muscle atrophy and lumbar spine flattening (Young et al., 2011; Chang et al., 2016), may adversely affect spinal functionality, potentially resulting in issues such as lower back pain (Pool-Goudzwaard et al., 2015) and increased risk of intervertebral disc (IVD) herniation post-flight (Johnston et al., 2010).

In fact, astronauts are considered to be four times more likely to suffer from disc herniation within the first-year post-mission compared to control groups (Johnston et al., 2010). The flattening of the spine in space is attributed to the absence of Earth’s gravitational load. In micro-gravity (micro-g), the spine is no longer compressed by this force, presumably leading to a relaxation of the muscles that normally support it. As a consequence, the spine appears to elongate and straighten, as evidenced by an 11% reduction in lumbar lordosis (flattening of the lumbar curve) immediately after returning from a long duration (approx. 6 months) ISS mission (Bailey et al., 2018). Similarly flattened lower lumbar lordosis was reported following a 60-day bedrest, whereas the upper lumbar spine exhibited increased lordotic curvature (Belavý et al., 2011).

In a study executed by our team, a reduction in lordosis, along with reduced trunk muscle activity and an increase in lumbar spinal stiffness, was observed even when the spine was exposed only briefly (approx. 22s) to micro-g during parabolic flight (Swanenburg et al., 2020). Studies on spinal curvature in varying gravity conditions have produced conflicting results. Initial findings suggested lumbar flattening during micro-g and hyper-gravity (hyper-g) phases (Swanenburg et al., 2020), but a later study failed to replicate these changes in hyper-g (Swanenburg et al., 2023). This discrepancy revealed limitations in sensor precision and coverage, indicating the need for more precise sensors and a larger array of sensors along the spine.

Furthermore, very few studies have investigated the thoracic or the transitional segments of the spine in response to gravitational change (Andreoni et al., 2000; de Martino et al., 2020; Somasundaram et al., 2021). This is unfortunate given that the transition segment between the relatively immobile thoracic, and the more flexible lumbar regions is known to be prone to injury (Somasundaram et al., 2021). Moreover, dry immersion (Treffel et al., 2017; Plehuna et al., 2022) and 8-hour hyper-bouyancy floatation (Green and Scott, 2018) studies have also revealed vertebral dysfunction and pain in the thoracolumbar region. However, the origin of the pain and the structures involved remain unknown. The concurrent measurement of extensor muscle activity and spinal curvature will provide insights into the underlying mechanisms as these muscles play a significant role in maintaining spinal curvature and stability (Hodges and Moseley, 2003). Indeed, changes in extensor activity may directly contribute to spinal curvature modulation, back pain, and risk of IVD herniation.

As a result, a comprehensive analysis of spinal curvature, including both the lumbar and thoracic regions, combined with an enhanced array of sensors in these regions is warranted. In addition, simultaneous measurement of extensor muscle activity at multiple points along the vertebral column might significantly contribute to our understanding of segmental spinal curvature adaptation. Such knowledge may inform the development of strategies to ameliorate in-flight back pain and attenuate post-flight disc herniation risk.

Therefore, the aim of this study is to evaluate spinal curvature across the lumbar and thoracic region, and extensor muscle activity during acute micro-g and hyper-g induced by parabolic flight. In addition, we investigated the association between our proxy measure of spinal curvature and extensor muscle activity in micro-g.

## 2 Methods

### 2.1 Participants and parabolic flight

18 healthy participants (8 females; mean age 33 ± 11 years) without low back pain participated in the parabolic flight study. Three male participants were experienced participants in parabolic flight missions. Participants completed the mandatory aviation medical screening, during which neurological and musculoskeletal disorders were ruled out (Ullrich and Buhler, 2019). Written informed consent was obtained from all participants before the study. Measurements were conducted during the European Space Agency (ESA) $80^{th}$ and $83^{rd}$ parabolic flight campaign (PFC) in Bordeaux, France. Both PFCs were operated by Novespace (Bordeaux, France) on board the Airbus A310 ZERO-G. The French “Comité de protection des personnes EST-III” approved the study (Nr-ID-RCB: 2022-A01696-37 21.07.2022).

### 2.2 Experimental design

**FIGURE 1:**
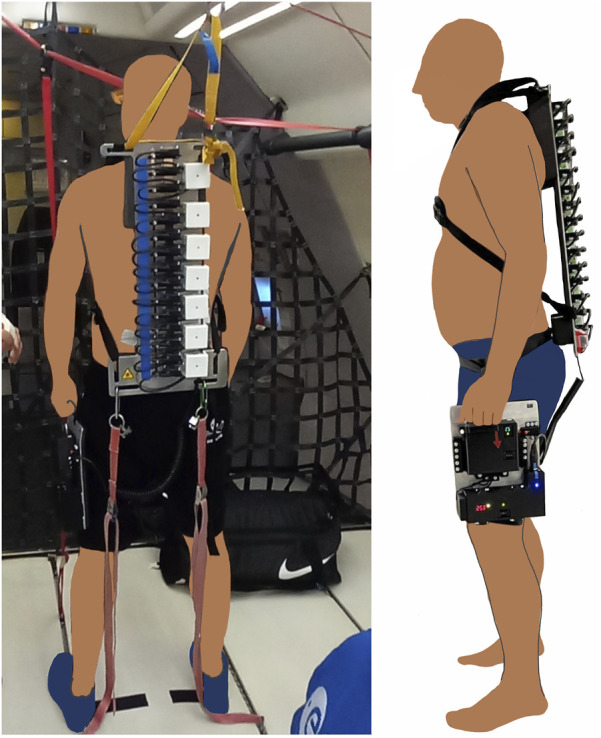
Measurement setup during PFC. Frontal and side view.

Spinal curvature and muscle activity were assessed at three gravitation levels (Gz): micro-g ( μ -Gz), earth-g (1.0-Gz), and hyper-g (approx. 1.8 Gz) induced by parabolic flight. Six flights were conducted during the two PFCs, each consisting of 31 parabolas (10/11 per participant). Each parabola began with horizontal flight at earth-g, subsequently transitioning into a steep ascent that resulted in hyper-g. The airplane then transitioned into micro-g as it pushed over the top of the parabola. Subsequently, a second phase of hyper-g followed during the return to level flight level. To mitigate potential motion sickness, each participant received a personalized dosage of scopolamine half an hour before the flight (Spinks and Wasiak, 2011; Ritzmann et al., 2016). To ensure participant safety, during parabolic flights, tethers connected the participant harness to the aircraft (Figure 1), preventing drifting in micro-g, or falling during hyper-g phases (Swanenburg et al., 2018).

### 2.3 Measurement setup

#### 2.3.1 Spinal curvature

Spinal curvature (combination of lumbar and thoracic) was evaluated using an array of fifteen optical distance sensors Class 1 laser (OM20-P0120.HH.YIN, Baumer Electric AG, Frauenfeld, Switzerland) affixed to two (long and short) aluminum back-plates mounted with a full-body harness. The sensors are designed to measure the distance to the participant’s skin with an accuracy of ± 0.05 mm and a repeatability of ± 0.01 mm. The small back-plate was used on two flights (6 participants) and the long back-plate on four flights (12 participants), depending on the trunk length of the participants. In either case the uppermost sensor was aligned with the height of cervical C7 with other sensors mounted 3 cm apart on the short back-plate and 3.5 cm on the long back-plate. Skin distances and acceleration were recorded continuously (effective average sampling rate of 1.26 Hz) during flight and transmitted to a laptop. In addition, the distance between the cervical C7 and the line connecting the two spina iliaca posterior superior (SIPS) was determined before the flight for each subject. These measurements were used to remove data from sensors positioned below the SIPS line from the analysis. The distance sensor array was powered by a 0.86 kg rechargeable battery (RS Pro NiMH Cs × 20 3200 mAh 24 V Pack, RS Components GmbH, Wädenswil, Swizerland), which was attached to a leg holster connected to the sensor array via a flexible cable to negate any impact on back alignment.

#### 2.3.2 Muscle activity

**FIGURE 2:**
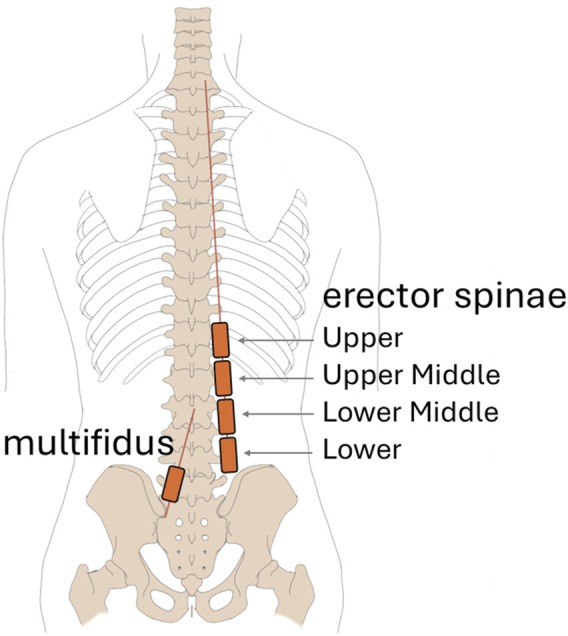
EMG transmitter placement.

Muscle activity of the erector spinae (ES) and the multifidus (MF) muscles were assessed with wireless surface electromyography (EMG) transmitters (pico/aktos; Myon AG, Schwarzenberg, Switzerland) equipped with integrated accelerometers (McGill et al., 1996; Jiroumaru et al., 2014). Preparation of each subject was in accordance with the Surface ElectroMyoGraphy for the Non-Invasive Assessment of Muscles (SENIAM) guidelines (Swanenburg et al., 2018). The pre-filtered EMG (2000 Hz, bandpass 10–500 Hz) and acceleration (148 Hz, bandpass 1–70 Hz) signals were captured by five transmitters. To gain information on ES activity across multiple sites, the first of four transmitters was attached on the right side at the level of L4 (ES lower) and three further transmitters (ES lower middle, ES upper middle, ES upper) were placed successively above (Figure 2). MF activity was assessed at the level of L5, with one transmitter placed on the left side.

### 2.4 Data preparation

All data was cleaned and aggreagted in MATLAB R2023a (MathWorks, 2023). Distance, electrical muscle activity (EMG), and acceleration data were recorded continuously during the flight. The acceleration signals were used for time-synchronizing distance and EMG data with continuous gravity recordings provided by the flight operator (Novespace), which were used for threshold-based segmentation into the different gravity phases as described in Supplementary Material S1.

#### 2.4.1 Spinal curvature

For the distance sensor array, the identified gravity segments were cut to windows of 17 s (. $25^{th}$ quantile of durations), and the mean distance was calculated within each window. In cases where female participants’ bra elastics interfered with a distance sensor, the mean value of the upper and lower adjacent sensors was used. The recordings of the upper most sensor was omitted due to intermittent data loss. Due to a temporary disruption of the recording system, data from two participants, loosing data from ten respectively three parabolas. Furthermore, one participant was affected by motion sickness for two parabolas, and thus, this data was discarded. In one participant, the posture moved in and out of the recording range for some of the sensors during the flight. Where this resulted in missing values for an entire gravity segment (per parabola), the affected sensors’ values were set to the sensors’ recording limits (16 mm min for sensors 8–10, 120 mm max for sensor 2).

The overall area captured between the backpack and the back was calculated as a proxy measure for spinal curvature. For comparison between gravity conditions, the data were normalized to the mean of all recordings for each participant to offset differences between the trunk heights of the participants. To correlate spinal curvature area and muscle activity during micro-g, the area was normalized to the area calculated for the participant in earth-g. The impact of repeated parabolas on spinal curvature was evaluated by comparing mean-normalized area (averaged across all gravity conditions) between the first, the last, and the average of all intermediate parabolas.

Greenhouse Geisser adjusted repeated measures ANOVA showed no significant difference between the parabolas (F (1.14, 15.98) = 0.15, p = 0.733). Therefore, data from all available parabolas was retained for further analysis.

In addition to area, we assessed spinal curvature via the angle formed between three adjacent sensors (inter-sensor angle). Inter-sensor angles of 180° indicate no curvature at that location, whereas angles <180° describe local lordosis, and angles >180° kyphosis.

#### 2.4.2 Muscle activity

ES and MF EMG records were time-synchronized with acceleration data provided by the flight operator by using the acceleration data recorded from the EMG transmitters. The resultant data was filtered using a second-order Butterworth filter with 10 and 500 Hz cutoff frequencies and rectified.

Following initial segmentation (Supplementary Material S1), all data segments were cut to the duration of the shortest recorded segment (14.86 s). Data segments that were affected by motion sickness were removed. Three times an EMG transmitter detached, resulting in data loss for all parabolas during those instances. In five cases, an EMG transmitter detached during recording, but sufficient data from multiple parabolas was retained for analysis.

EMG root mean square was calculated for each segment. To determine validity, muscle activity was compared between the averaged segments of the first, the last and the mean of all other parabolas. Outliers [beyond 3 * inter quartile range from the first and third quartile (Kassambara, 2023)] were detected and the data was not normally distributed (Shapiro-Wilk Test). Therefore, robust repeated measures ANOVA (Mair and Wilcox, 2020) was used. There was a significant difference between parabolas for ES transmitters 1, 3 and 4, and the MF transmitter. Based on these results, data from the first parabola was removed from the analysis of the EMG data (Supplementary Material S2).

EMG data for each transmitter was normalized to the mean value of all segments of each participant across all gravity conditions. Thus, values of 100% reflect the average EMG activity on the corresponding sensor for a person, independently of the assessed gravity condition. For correlation (with spinal curvature data in micro-g), EMG data was normalized to the mean of the earth-g conditions of each participant.

### 2.5 Data analysis

Data was analyzed in R (R Core Team, 2023) with statistics calculated using the R package rstatix (Kassambara, 2023). Following confirmation of normality using Shapiro-Wilk tests and testing for the presence of extreme outliers (beyond 3 * inter quartile range from the first and third quartile (Kassambara, 2023)) within each gravity condition, one-way repeated measures ANOVA was used to compare mean-normalized area between the gravity conditions with Post-hoc Bonferroni-corrected pairwise t-tests. Post hoc power analysis was performed for the *post hoc* comparisons using G ∗ Power (Faul et al., 2007) Version 3.1.9.7.

ES EMG activity was compared between gravity conditions separately for each EMG sensor by using Friedman tests with Bonferroni correction. Post-hoc comparisons were performed (if indicated) via Bonferroni-corrected Wilcox signed-rank tests. MF activity was compared across gravity conditions by using Friedman test and Wilcox signed-rank testing after the assumption of normality was rejected for the earth-g condition.

In addition, the association between muscle activity and spinal curvature was investigated for the transition to micro-g with all data normalized to earth-g. Spearman (ρ) was used, as Shapiro-Wilk tests indicated that the data was not normally distributed.

## 3 Results

### 3.1 Spinal curvature

**FIGURE 3:**
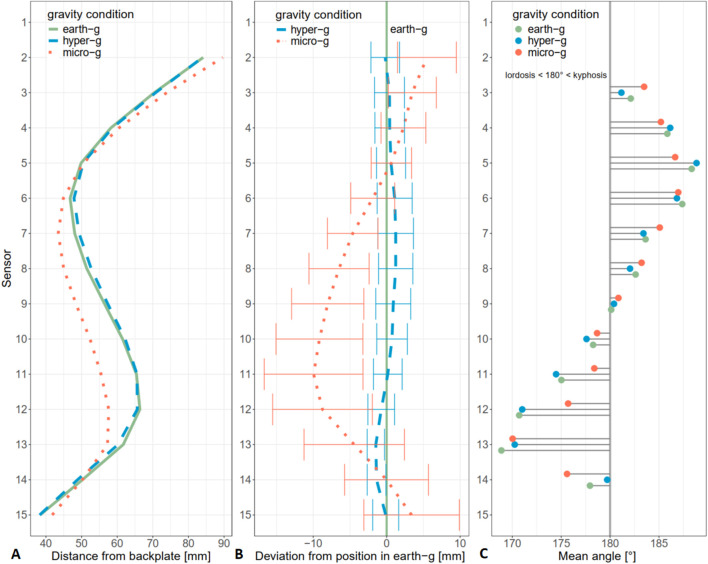
**(A)** Mean distance from backplate under different gravity conditions per sensor. **(B)** Mean deviation from earth-g during hyper-g and micro-g per sensor. Error bars show standard deviations. **(C)** Mean angles between adjacent sensors. Angles of 180° indicate no curvature. Smaller angles indicate a lordotic, larger angles a kyphotic alignment.

There was a significant (F (1.19, 19.02) = 22.45, p < 0.001) effect of gravity level on the mean-normalized area (earth g: M = 101.9, SD = 1.9; hyper-g: M = 102.4, SD = 2.2; micro-g: M = 95.7, SD = 3.6). Post-hoc tests showed no difference between earth-g and hyper-g (t (16) = −0.94, p = 1.00). However, the mean-normalized area was significantly smaller in micro-g vs. both earth-g (t (16) = 4.84, p = 0.001) and hyper-g (t (16) = 4.91, p = 0.001) indicative of spinal flattening. Post-hoc power analysis indicated sufficient power for detecting differences between micro-g and earth-g (97%) and micro-g and hyper-g (98%), but not for differences between earth-g and hyper-g (8%) in mean-normalized area. Inspection of the sensor data revealed that the area reduction occurred between the $7^{th}$ and $12^{th}$ sensor (Figures 3A,B). Spinal curvature described by inter-sensor angle (Figure 3C), suggested a “flattened” lordotic angle (closer 180°), at sensor 11 (earth-g: M = 175.0°, SD = 3.2°; micro-g: M = 178.4°, SD = 3.8°) and sensor 12 (earth-g: M = 170.7°, SD = 5.3°; micro-g: M = 175.7°, SD = 4.0°) during micro-g.

### 3.2 Muscle activity

#### 3.2.1 Erector spinae activity

**FIGURE 4:**
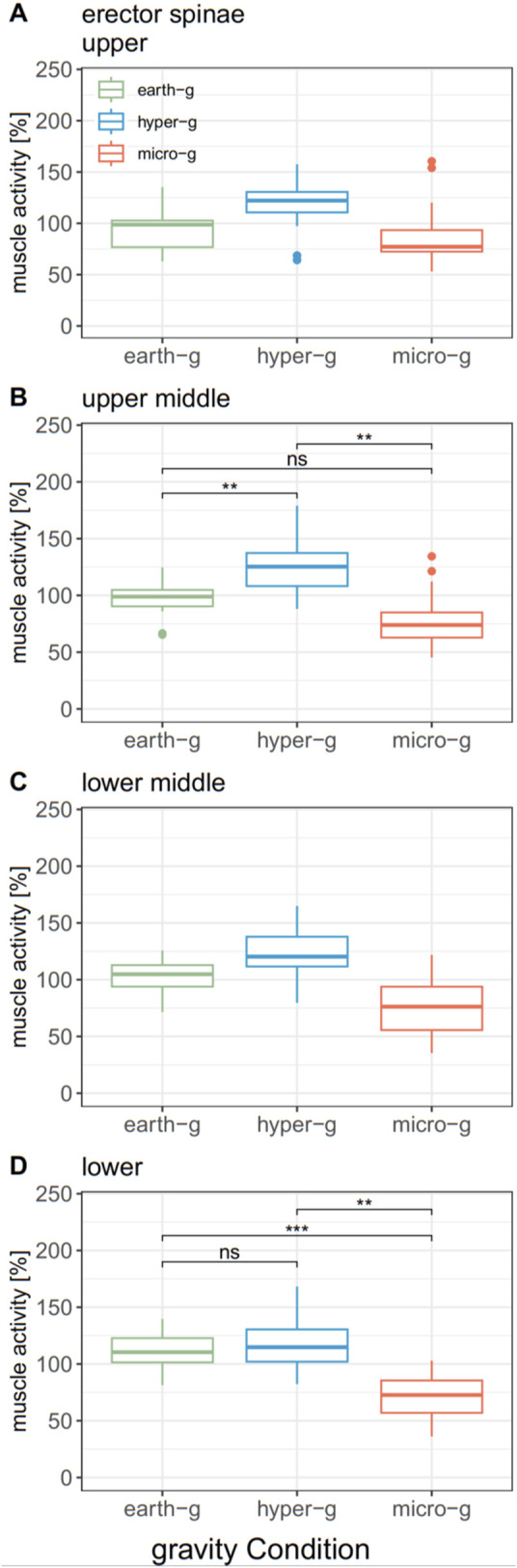
Comparison of mean normalized erector spinae activity at different transmitter positions between gravity conditions. **(A)** Erector spinae activity at the upper transmitter, **(B)** Erector spinae activity at the upper middle transmitter, **(C)** Erector spinae activity at the lower middle transmitter, **(D)** Erector spinae activity at the lower transmitter. ∗∗p<.01;∗∗∗p<.001

The gravity level significantly affected ES muscle activity at the lower ES transmitter (L4) and upper middle, but not lower middle and upper (Tables 1, 2). Post-hoc tests indicate that lower ES muscle activity was significantly reduced in micro-g vs. earth-g and hyper-g (Table 3; Figure 4D). In contrast, the upper middle ES transmitter muscle activity was significantly higher in hyper-g, compared to the other conditions (Table 3; Figure 4B).

**TABLE 1:** Means and standard deviations of mean normalized muscle activity for different gravity segments.

Assessment	Participants	Earth-g	Hyper-g	Micro-g
	*n*	$M^{c}$ ± ${SD}^{d}$	***M*** ± *SD*	*M* ± *SD*
gravity $\left[m/s^{2}\right]$	16	1.00 ± 0.01	1.79 ± 0.01	−0.00 ± 0.00
${ES}^{a}$ upper [%]	16	92.19 ± 18.71	119.16 ± 26.06	88.64 ± 31.28
ES upper middle [%]	16	96.63 ± 15.11	124.60 ± 23.70	78.77 ± 25.26
ES lower middle [%]	16	103.26 ± 15.66	120.81 ± 23.16	75.93 ± 25.83
ES lower [%]	14	111.64 ± 14.87	117.52 ± 22.11	70.84 ± 18.63
${MF}^{b}$ [%]	15	113.34 ± 14.57	122.53 ± 24.32	64.14 ± 30.46

${Note.}^{a}$ Erector ${\text{Spinae;}}^{b}$ ${\text{Multifidus;}}^{c}$ ${\text{Mean;}}^{d}$ Standard Deviation.

**TABLE 2:** Comparison in mean normalized Erector Spinae activity [%] between gravity conditions for different sensors.

Transmitter position	*n*	$\chi^{2}$	*df*	*p*- ${\text{value}}^{a}$
ES upper	16	6.50	2	0.155
ES upper middle	16	11.38	2	**0.014**
ES lower middle	16	8.00	2	0.073
ES lower	14	15.43	2	**0.002**

${Note.}^{a}$ *p*-values are Bonferroni corrected.

**TABLE 3:** Post-hoc comparison of mean normalized Erector Spinae activity [%] between gravity conditions.

Transmitter position	Comparison	*W*	*p*- ${\text{value}}^{a}$
ES upper middle	earth-g vs.hyper-g	11	**0.005**
ES upper middle	earth-g vs. micro-g	106	0.152
ES upper middle	hyper-g vs. micro-g	125	**0.005**
ES lower	earth-g vs. hyper-g	44	1
ES lower	earth-g vs. micro-g	104	< **.001**
ES lower	hyper-g vs. micro-g	103	**0.001**

${Note.}^{a}$ *p*-values are Bonferroni corrected.

#### 3.2.2 Multifidus activity

**FIGURE 5:**
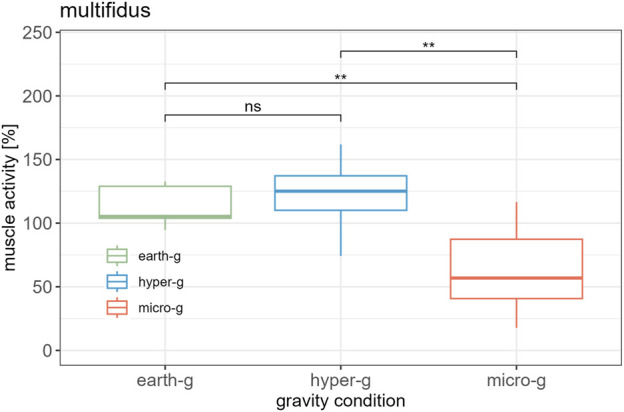
Comparison of mean normalized multifidus activity between different gravity conditions. ∗∗p<.01;∗∗∗p<.001

A significant effect of gravity level on MF muscle activity was observed at L5 ( $\chi^{2}$ (2) = 12.4, p = 0.002). Post-hoc tests showed no significant difference between earth-g and hyper-g (W = 39, p = 0.756). However, MF activity was significantly lower in micro-g compared to both earth-g (W = 114, p = 0.003) and hyper-g (W = 112, p = 0.005) (Figure 5).

### 3.3 Association between spinal curvature and muscle activity during micro-g

There was a positive association between earth-normalized muscle activity and earth-normalized area at the lower ES (L4) level during micro-g, but not for the other 3 ES transmitters. There was also a positive association between earth-normalized area and MF (L5) muscle activity (Table 4).

**TABLE 4:** Correlation between area measures and muscle activity normalized to earth-g.

Muscle, transmitter	$n^{c}$	$\rho^{d}$	***p-value*** ^e^
${ES}^{a}$ upper [%]	15	0.25	0.750
ES upper middle [%]	15	0.51	0.105
ES lower middle [%]	15	0.39	0.313
ES lower [%]	13	0.67	**0.030**
${MF}^{b}$ [%]	14	0.69	**0.004**

${Note.}^{a}$ ES = Erector ${\text{Spinae;}}^{b}$ MF = ${\text{Multifidus;}}^{c}$ complete ${\text{cases;}}^{d}$ Spearman correlation ${\text{coefficient;}}^{e}$ *p*-values of the Erector Spinae assessments are Bonferroni corrected.

## 4 Discussion

The study’s main findings were that even acute exposure to micro-g significantly modulated spinal curvature with flattening of the upper lumbar and lower thoracic spine. Spinal flattening was evident both as cumulative area, and inter-sensor angle derived from a novel optical distance sensor array. Multifidus (MF) activity at L5 and Erector Spinae (ES) activity at L4 were also reduced in micro-g, but no other muscle activity changes were observed. Increased ES activity was observed at the upper middle transmitter during hypergravity. Significant positive correlations were observed between spinal flattening in micro-g and both MF activity at L5, and lower ES activity (L4).

### 4.1 Spinal curvature

#### 4.1.1 Micro-g

The cumulative area, and inter-sensor angle measurements based on a novel optical distance array revealed rapid and reversible spinal flattening in response to micro-g induced during parabolic flight. Previous research has reported flattening of the spinal lumbar lordosis following post-long-duration space missions (Bailey et al., 2018). While our study measured immediate spinal adaptations in standing position during parabolic flights, Bailey et al. (2018) observed similar patterns of lumbar flattening after long-duration spaceflight using supine MRI positioning, a posture that naturally induces some spinal flattening, with post-mission measurements showing even greater flattening compared to pre-mission baseline, suggesting these adaptations represent fundamental physiological responses to microgravity that manifest immediately and persist or potentially worsen regardless of measurement position or exposure duration. However, the time course, and mechanisms underlying lumbar flattening are unknown (Green and Scott, 2018). Spinal tissue, including ligaments and IVDs are reported to deteriorate in-flight, with a range of IVD pathologies reported with in-flight ultrasound (Garcia et al., 2018). Such changes may contribute to reported post-flight spinal issues such as IVD prolapse (Johnston et al., 2010).

The optical sensor array identified the upper lumbar and lower thoracic spine as areas that account for the majority of micro-g induced flattening. This contrasts with the hypothesis that the lower lumbar spine is the most affected by micro-g as it typically bears the highest (cumulative) load (Belavý et al., 2011). However, our findings may explain why approx. 60% of all spinal injuries in astronauts found in post-flight MRI occur in the upper lumbar spine (Bailey et al., 2022).

The curvature flattening we report is rapidly induced but appears consistent with changes in lumbar geometry and kinematics observed post-flight (Bailey et al., 2022) and in 4 hours of lying on a ground-based analogue of micro-g; hyper-buoyancy floatation (HBF) (Breen et al., 2023). It may also be consistent with, or even a factor in the generation of complex vertebral stiffness changes reported in PFCs (Swanenburg et al., 2020) and 4 hours of HBF unloading (Marcos-Lorenzo et al., 2024) across the spine. Interestingly, in the latter study, reloading did not reverse micro-g changes, suggesting that unloading may generate persistent vertebral vulnerability.

#### 4.1.2 Hyper-g

Hyper-g spinal curvature measures were observed to not differ from earth-g. Interestingly, our earlier studies using two ultrasonic distance sensors yielded contradictory spinal curvature changes during hyper-g conditions (Swanenburg et al., 2020; Swanenburg et al., 2023). As a result, we evaluated this disparity by employing a bespoke array of 15 optical instead two less accurate ultrasonic distance sensors (Swanenburg et al., 2020; Swanenburg et al., 2023). Thus, we believe that the changes previously reported may be artefactual.

Whilst 1.8 g did not induce significant spinal curvature modifications compared to 1 g, the data suggests it rapidly ‘corrects’ micro-g-induced spinal changes. Interestingly, whilst spinal curvature is restored - there are notable changes in spinal motor activity during hyper-g.

### 4.2 Muscle activity

#### 4.2.1 Erector spinae

Our multi-transmitter EMG analysis revealed differential ES muscle responses along the spinal axis. During micro-g conditions, a significant reduction in muscle activity was observed at the lower ES transmitter (L4), while earth-g and hyper-g conditions demonstrated similar muscle activation patterns. Conversely, the upper middle ES muscle exhibited increased activity during hyper-g exposure, significantly greater than both earth-g and micro-g conditions. Bedrest studies have reported that ES and MF muscle atrophy exhibits significant inter-muscular and segmental variations (Belavý et al., 2011). Similarly, post-flight investigations suggest that MF muscle atrophy is particularly pronounced in the lower lumbar region, highlighting the need for a nuanced segment-specific approach when evaluating spinal muscle responses (Bailey et al., 2022; Hides et al., 2021). During earth-g and hyper-g conditions, the lower lumbar muscular segments actively engage to stabilize the body’s upright posture. Notably, hyper-g conditions revealed additional paraspinal muscular activation in the upper lumbar and lower thoracic spine, starkly contrasting with the muscular inactivation observed in the lower spinal region during micro-g exposure. These differential activation patterns suggest that muscular stabilization mechanisms during hyper-g are robust and maintain spinal structural integrity without inducing significant curvature alterations.

#### 4.2.2 Multifidus

In our study, activation of the MF at L5 decreased in micro-g but did not differ in response to hyper-g compared to 1 g. This pattern is similar to that which we observed for the ES muscle at L4. A reduction of MF activity in micro-g is consistent with research that has demonstrated MF muscle atrophy following unloading induced by bed-rest (Belavý et al., 2011) and spaceflight (Bailey et al., 2022; Hides et al., 2021). However, in a previous parabolic flight experiment, no significant differences in MF activity were observed in response to various levels of hypogravity (de Martino et al., 2020). Yet, response to trunk perturbations was attenuated - with attenuation increasing as g levels reduced (de Martino et al., 2020). Such changes may underpin the associations reported between MF atrophy and low back pain in bed rest (Belavý et al., 2011; Ranger et al., 2017), and inter-vertebral disc herniation post spaceflight (Bailey et al., 2022).

### 4.3 Interaction between spinal curvature and muscle activity

Our analysis revealed significant correlations between earth-normalized area during micro-g conditions and muscle activity of the lower ES at L4, and MF at L5. These findings indicate a direct relationship between spinal curvature flattening and reduced muscle activity at these lower spinal segments.

A relationship between spinal curvature flattening and reduced ES (L4) and MF (L5) muscle activity is consistent with the research in astronauts by Bailey and colleagues (Bailey et al., 2018), who demonstrated a strong association between lumbar flattening, MF muscle atrophy, and increased spinal stiffness. On earth, such biomechanical alterations are typically associated with compromised spinal mechanics and frequently low back pain (Matheve et al., 2023). Consequently, these changes may substantially elevate the risk of injury upon return to gravitational conditions, particularly in individuals with pre-existing vertebral end plate insufficiency.

Injury risk is likely linked to changes in spinal biomechanics. Complex, level dependent changes in spinal biomechanics have been reported in response to spaceflight (Bailey et al., 2018) and following a 4 h of HBF via fluoroscopy (Breen et al., 2023). Whilst sensitive, these measures are not compatible with spaceflight whilst ‘spinal stiffness’ – which can be measured with a small hand-held devices has been proposed as a proxy measure for motor control contribution to spinal stability (Hofstetter et al., 2018).

Spinal stiffness has demonstrated nuanced changes in spinal stabilization strategies under hyper-g conditions (Swanenburg et al., 2018; Swanenburg et al., 2020). Large-scale ground-based investigations using axial loading have confirmed the modulation of spinal stabilizers (Häusler et al., 2020; Glaus et al., 2021) although the picture post-HBF unloading is unclear (Marcos-Lorenzo et al., 2024). These findings collectively underscore the complex and dynamic adaptive capabilities of the musculoskeletal system when subjected to acute changes in axial loading. One hypothesis is that increases in spinal stiffness during hyper-g can be attributed to the mechanical response of abdominal muscle activation under additional axial loading (Bergmark, 1989) that attempts to facilitate load redistribution, including ‘load-sharing’ with the thoracic cage and pelvis (Bergmark, 1989; Swanenburg et al., 2023). The apparent contradiction between stable spinal curvature and dynamic spinal stabilization strategies reveals the intricate neuromuscular adaptability of the human spine in response to altered gravitational conditions.

### 4.4 Other considerations and implications

This study demonstrates that even brief exposures to micro-g, lasting about 20 s, can induce temporary flattening mainly of the upper lumbar and lower thoracic spine. The rapid spinal adaptations we observed during PFC short-duration micro-g exposure raise important questions about the cumulative effects of long-duration space missions on spinal health. An unanswered question is how long it takes for the motor control of the spine to return to its original state after exposure to altered gravity.

Flattening of the upper lumbar and lower thoracic spine initially results in frontal extension and potential contraction of dorsal ligaments (Panjabi et al., 1982). While these structures maintain their integrity at the beginning of a micro-g mission, extended exposure significantly degrades tissue quality (Chang et al., 2016; Moosavi et al., 2021; Bailey et al., 2022). Upon re-exposure to Earth’s gravitational forces, these structures must re-adapt, whilst under 1 g loading but compounded by the micro-g induced degradation of passive structural integrity, potentially increasing the risk of injury or dysfunction.

The biomechanical flattening of lumbar lordosis during micro-g exposure reveals critical implications for musculoskeletal dynamics, particularly concerning the psoas muscle. As the lordosis flattens, the psoas muscle, which attaches to the anterior sides of the lumbar vertebral bodies, is placed in an elongated position (Jorgensson, 1993). In a micro-g environment, this muscle elongation, combined with the absence of axial load, might lead to its constant activation. While most back muscles experience decreased activity and mass degeneration during extended space missions (Chang et al., 2016; LeBlanc et al., 2000), the psoas muscle uniquely maintains its size and may even increase its activity (Andreoni et al., 2000; Penning 2000 suggested that the psoas adjusts its activity based on the degree of lumbar curvature in micro-g, which may contribute to this persistent activation (Swanenburg et al., 2020; Penning, 2000). The constant involvement of the psoas in this elongated state could cause muscle fatigue and lumbar pain (Granata et al., 2004; Johnston et al., 1998; Barker et al., 2004), which may explain the low back pain experienced by some astronauts early in their missions (Johnston et al., 2010). Adopting a fetal “tuck” position in weightlessness can provide relief (Penning, 2000), probably by returning the psoas muscle to a more normal length. These findings support the hypothesis that flattening of the spine and subsequent changes in psoas muscle function is central to the development of back pain in astronauts.

Our segmental EMG analysis revealed heterogeneous acute responses along the ES muscle, emphasizing the importance of considering regional variations in muscle activity when studying spinal adaptations to altered gravity. This finding suggests that single-site EMG measurements are inadequate to capture the complexity of trunk muscle responses to gravitational changes. Future studies should consider multi-site (per muscle) or high-density EMG techniques. Understanding changes in motor control of the spine under varying gravitational loads may also explain changes in proprioception (Swanenburg et al., 2023), which plays a crucial role in promoting posture, balance, and coordinated movement (Proske and Gandevia, 2012). Whilst peripheral proprioception has been evaluated as a risk factor for crew falls or impaired performance (Bloomberg et al., 2015). Less attention has been paid to the role of trunk (core) proprioception.

The results reported in this manuscript may have implications for spacesuit and spacecraft seat design. The immediate spinal adaptations observed during microgravity exposure suggest that such equipment might benefit from considering dynamic support elements and modified curvature profiles. Additionally, approaches that potentially promote active muscle engagement during movement could possibly help maintain proper spinal function and might reduce the risk of injuries during gravitational transitions.

### 4.5 Limitations and strengths

Although our methodology allowed us to investigate the effects of micro-g on spinal curvature more comprehensively and beyond the lumbar spinal segment, no exact mapping of anatomical landmarks with the recorded data was possible and should be considered for future experiments. Although the sensor array was fitted to the participants with backpack straps and secured at the hip, potential shifts of the backpack were not measured and cannot be excluded. Large standard deviations were observed at the lowest sensors for angles calculated from distance data. These may be influenced by clothing having moved into the sensor’s fields. Regarding the EMG data, the overall muscular activity was low due to the static assessment posture. Therefore, the EMG sensors captured additional electric activity from the heart, which could not be removed from the signal. However, the impact of this crosstalk effect was deemed minor. In addition, potential rapid fluid displacements are unlikely to having affected the results (Von Walden et al., 2008).

## 5 Conclusion

In conclusion, brief changes in gravity induced by parabolic flight significantly modulated spinal curvature with flattening of the upper lumbar and lower thoracic spine observed in response to micro-g, but no curvature change in hyper-g. Multifidus at L5 and Erector Spinae muscle activity at L4 were also reduced in micro-g, but no other muscle activity changes were observed. Increased ES activity was observed at the upper middle transmitter during hyper-g. The maintained spinal curvature combined with targeted muscle activation during hyper-g demonstrates the spine’s natural protective mechanisms against increased axial loading. This robustness is crucial for maintaining posture and preventing injury during exposure to increased axial loading and is key to understanding spinal health in terrestrial and space environments. The observed changes in spinal curvature and segmental muscle activity patterns in micro-g underscore the need for targeted countermeasures to maintain spinal health during spaceflight.

## Data Availability

The raw data supporting the conclusions of this article will be made available by the authors, without undue reservation.
