# Supplementary material for: Adaptation of thoracic and lumbar curvature and spinal muscle activity under changing gravity
Source: Front Physiol. 2025 May 20;16:1549249. doi: 10.3389/fphys.2025.1549249 (PMC12131012; doi:10.3389/fphys.2025.1549249)
Supplement: Supplementary file 1 [file DataSheet2.pdf]

frontiers

Supplementary Material 2

Comparison between EMG sensor data between the first parabola assessed for each participant, the last parabola and the average of all other parabolas.

Table S1. Normality (Shapiro-Wilk test) and Sphericity (Mauchly's test) assumption tests for comparisons between parabolas

	Normality						Sphericity		
	First- parabolas		Center- parabolas		Last -Parabolas				
Outcome	W	p-value	W	p-value	W	p-value	W	p-value	ϵ
mean-normalized area	.980	.970	.956	.631	.963	.736	0.247	**<.001**	.57
ES *upper*	.898	.074	.986	.995	.916	.169			
ES *upper middle*	.790	**.002**	.878	**.036**	.890	.081			
ES *lower middle*	.717	**<.001**	.822	**.005**	.821	**.005**			
ES *lower*	.891	.082	.983	.988	.817	**.008**			
MF	.809	**.005**	.738	**<.001**	.976	.943			
	Earth-g		Hyper-g		Micro-g				
mean-normalized area	.912	.108	.946	.392	.938	.292	0.318	**<.001**	.60
ES *upper*	.926	.211	.918	.157	.823	**.006**			
ES *upper middle*	.925	.206	.959	.649	.902	.086			
ES *lower middle*	.962	.706	.969	.836	.971	.858			
ES *lower*	.972	.901	.952	.585	.975	.938			
MF	.841	**.013**	.982	.980	.953	.569			

Table S2. Comparison of muscle activity between the first parabola, the last parabola and mean of the center parabolas.

Muscle, sensor number	F	df1	df2	p-value
ES *upper*	4.99	1.77	14.20	**.026**
ES *upper middle*	6.00	1.44	12.98	**.021**
ES *lower middle*	3.00	1.60	14.42	.090
ES *lower*	11.30	1.55	13.99	**.002**
MF	19.53	1.27	11.40	**.001**

Note. Results printed in bold are accepted as statistically significant.

Table S3. Post hoc tests comparing muscle activity between the first parabola, the last parabola and mean of the center parabolas.

Muscle, sensor number	comparison	$\hat{\psi}$	p-value	p-value cutoff
ES *upper*				
	first vs. center	13.22	.053	.025
	first vs. last	12.48	.035	.017
	center vs. last	2.42	.542	.050
ES *upper middle*				
	first vs. center	17.60	**.005**	.017
	first vs. last	19.61	.077	.025
	center vs. last	-0.02	.997	.050
ES *lower*				
	first vs. center	14.00	.045	.025
	first vs. last	19.52	.043	.017
	center vs. last	6.09	.073	.050
MF				
	first vs. center	32.89	**.006**	.025
	first vs. last	45.07	**.003**	.017
	center vs. last	10.48	.112	.050

Note. Results printed in bold are accepted as statistically significant.

Table S4. Shapiro-Wilk test results for earth normalized data in micro-g.

Outcome	W	p-value
Earth-normalized area	.934	.309
Earth-normalized *ES upper*	.871	**.035**
Earth-normalized ES *upper middle*	.822	**.007**
Earth-normalized ES *lower middle*	.950	.525
Earth-normalized ES *lower*	.982	.989
Earth-normalized MF	.896	.099
