# Supplementary material for: Adaptation of thoracic and lumbar curvature and spinal muscle activity under changing gravity
Source: Front Physiol. 2025 May 20;16:1549249. doi: 10.3389/fphys.2025.1549249 (PMC12131012; doi:10.3389/fphys.2025.1549249)
Supplement: Supplementary file 2 [file DataSheet1.pdf]

Supplementary Material 1

1 SEGMENTATION INTO GRAVITY PHASES

Gravity data was filtered using a moving average of 9 samples and automatically segmented into 5 gravity phases per parabola. Two earth gravity segments, two hyper gravity segments and a micro gravity segment were initially extracted, but data from the second hypergravity segment and second earth gravity segment were not further analyzed as the second hypergravity segment was shorter and the gravity conditions appeared less stable. Hypergravity was defined as the time between a threshold-based onset and endpoint. The onset of hypergravity was detected as the first sample exceeding a threshold of 1.5 g and the onset of a new hypergravity segment could be detected earliest after a latency period of 25 seconds. The end of hypergravity was determined according to the same pattern but based on the reversely ordered data. Detected onsets and endpoints less than 2 seconds apart were discarded. Microgravity was defined analogously with gravity falling below a threshold of 0.3 g. The onset of earth gravity was defined to begin 24 seconds before hypergravity and to last 20 seconds. Appendix 1: Segmentation into Gravity phases Gravity data was filtered using a moving average of 9 samples and automatically segmented into 5 gravity phases per parabola. Two earth gravity segments, two hyper gravity segments and a micro gravity segment were initially extracted, but data from the second hypergravity segment and second earth gravity segment were not further analyzed as the second hypergravity segment was shorter and the gravity conditions appeared less stable. Hypergravity was defined as the time between a threshold-based onset and endpoint. The onset of hypergravity was detected as the first sample exceeding a threshold of 1.5 g and the onset of a new hypergravity segment could be detected earliest after a latency period of 25 seconds. The end of hypergravity was determined according to the same pattern but based on the reversely ordered data. Detected onsets and endpoints less than 2 seconds apart were discarded. Microgravity was defined analogously with gravity falling below a threshold of 0.3 g. The onset of earth gravity was defined to begin 24 seconds before hypergravity and to last 20 seconds.
